# Chemosensitizing effect of pentoxifylline in sensitive and multidrug-resistant non-small cell lung cancer cells

**DOI:** 10.20517/cdr.2024.04

**Published:** 2024-05-20

**Authors:** Beatriz S. Matos, Sara Peixoto da Silva, M. Helena Vasconcelos, Cristina P. R. Xavier

**Affiliations:** ^1^i3S - Instituto de Investigação e Inovação em Saúde, Universidade do Porto, Rua Alfredo Allen 208, Porto 4200-135, Portugal.; ^2^Cancer Drug Resistance Group, IPATIMUP - Institute of Molecular Pathology and Immunology of the University of Porto, Rua Alfredo Allen 208, Porto 4200-135, Portugal.; ^3^Department of Biological Sciences, FFUP - Faculty of Pharmacy, University of Porto, Rua de Jorge Viterbo Ferreira 228, Porto 4050-313, Portugal.; ^4^UCIBIO - Applied Molecular Biosciences Unit, Toxicologic Pathology Research Laboratory, University Institute of Health Sciences (1H-TOXRUN, IUCS-CESPU), Gandra 4585-116, Portugal.; ^5^Associate Laboratory i4HB - Institute for Health and Bioeconomy, University Institute of Health Sciences - CESPU, Gandra 4585-116, Portugal.

**Keywords:** Chitinase 3-like-1, combined therapies, drug repurposing, multidrug resistance, non-small cell lung cancer, pentoxifylline

## Abstract

**Aim:** Multidrug resistance (MDR) is frequent in non-small cell lung cancer (NSCLC) patients, which can be due to its fibrotic stroma. This work explores the combination of pentoxifylline, an anti-fibrotic and chitinase 3-like-1 (CHI3L1) inhibitor drug, with conventional chemotherapy to improve NSCLC treatment.

**Methods:** The effect of pentoxifylline in the expression levels of P-glycoprotein (P-gp), CHI3L1 and its main downstream proteins, as well as on cell death, cell cycle profile, and P-gp activity was studied in two pairs of sensitive and MDR counterpart NSCLC cell lines (NCI-H460/NCI-H460/R and A549/A549-CDR2). Association studies between *CHI3L1* gene expression and NSCLC patients’ survival were performed using The Cancer Genome Atlas (TCGA) analysis. The sensitizing effect of pentoxifylline to different drug regimens was evaluated in both sensitive and MDR NSCLC cell lines. The cytotoxicity of the drug combinations was assessed in MCF10A non-tumorigenic cells.

**Results:** Pentoxifylline slightly decreased the expression levels of CHI3L1, β-catenin and signal transducer and activator of transcription 3 (STAT3), and caused a significant increase in the G1 phase of the cell cycle in both pairs of NSCLC cell lines. A significant increase in the % of cell death was observed in the sensitive NCI-H460 cell line. TCGA analysis revealed that high levels of CHI3L1 are associated with low overall survival (OS) in NSCLC patients treated with vinorelbine. Moreover, pentoxifylline sensitized both pairs of sensitive and MDR NSCLC cell lines to the different drug regimens, without causing significant toxicity to non-tumorigenic cells.

**Conclusion:** This study suggests the possibility of combining pentoxifylline with chemotherapy to increase NSCLC therapeutic response, even in cases of MDR.

## INTRODUCTION

Despite recent advances in the treatment of non-small cell lung cancer (NSCLC), platinum-based chemotherapy, consisting of cisplatin or carboplatin in combination with paclitaxel, gemcitabine or vinorelbine, is still commonly used^[1-5]^. Nevertheless, multidrug resistance (MDR) to chemotherapy is frequently observed in patients with NSCLC^[6,7]^. A high expression of efflux pumps, particularly P-glycoprotein (P-gp), as well as an overexpression of constitutive androstane receptor (CAR), which is a positive regulator of P-gp expression, has been associated with MDR^[8-11]^. Importantly, the presence of a highly fibrotic stroma in patients with NSCLC, which hampers therapeutic efficacy by preventing drugs from reaching tumor cells, also contributes to MDR^[12-14]^. Thus, therapeutic approaches based on the combination of conventional chemotherapy with anti-fibrotic drugs might improve the treatment outcome of NSCLC patients.

Our recent work discovered that pentoxifylline, an anti-fibrotic drug approved for the treatment of vascular diseases^[15,16]^, increased the response of pancreatic cancer cells to gemcitabine treatment^[17]^. The chemosensitizing effect of pentoxifylline has also been described in cervical^[18,19]^, prostate^[20]^, and human pancreatic tumor xenografts^[21]^. In NSCLC cells, Ohsaki *et al.* reported the benefit of combining pentoxifylline with cisplatin and etoposide by interfering with the cell cycle^[22]^. Thus, pentoxifylline, in combination therapies, may be a promising drug candidate for drug repurposing to improve NSCLC treatment. Mechanistically, pentoxifylline showed anticancer activity by reducing tumorigenesis and cell proliferation via modulation of the mitogen-activated protein kinase/extracellular signal-regulated kinase (MAPK/ERK) and protein kinase B (Akt) pathways^[23]^, and inducing apoptosis and cell cycle arrest in breast^[24]^, liver^[25]^, and colorectal^[26]^ cancer cells.

Interestingly, pentoxifylline is also known as a chitinase 3-like-1 (CHI3L1) inhibitor^[17,27]^. Notably, CHI3L1 is a promising therapeutic target in cancer. For instance, CHI3L1 has been implicated in reducing the response of pancreatic cancer cells to gemcitabine^[17]^. Several studies have also reported that high serum levels of CHI3L1 are associated with low overall survival (OS) rates in patients with different types of cancer^[28-30]^. In NSCLC, Jefri *et al.* demonstrated that CHI3L1 mediates the expression of numerous epithelial-to-mesenchymal transition factors, providing an invasive phenotype to cancer cells^[31]^. CHI3L1 is associated with tissue remodeling through stimulation of fibroblast growth and matrix deposition, thus contributing to establishing a highly fibrotic stroma in NSCLC^[32,33]^. CHI3L1 is also known to be secreted by tumor and stromal cells, such as macrophages and fibroblasts^[33]^. Most importantly, CHI3L activates several pro-survival signaling pathways, and regulates cell growth, differentiation, and apoptosis in NSCLC. For instance, CHI3L1 interacts with IL-13Rα2 and/or the receptor for advanced glycation end products (RAGE), which are transmembrane receptors, activating ERK, Akt, and signal transducer and activator of transcription 3 (STAT3) signaling pathways, and inducing β-catenin nuclear translocation^[33-36]^. Thus, inhibitors of CHI3L1 may be promising drugs to be repurposed, in order to be used in NSCLC treatment in combination with chemotherapy.

Therefore, this work intends to disclose the mechanism of action of pentoxifylline and to explore the chemosensitizing effects of pentoxifylline when combined with conventional chemotherapeutic drugs currently used to treat NSCLC (paclitaxel, vinorelbine, carboplatin, and vinorelbine plus carboplatin), in pairs of sensitive and MDR NSCLC cell lines.

## METHODS

### Cell lines and culture

Two pairs of counterpart NSCLC cell lines, NCI-H460 (sensitive) and NCI-H460/R (MDR) and A549 (sensitive) and A549-CDR2 (MDR), were used in this work. The human NSCLC large cell carcinoma NCI-H460 and NCI-H460/R cell lines were kindly provided by Dr. Milica Pešić, Belgrade, Serbia^[37,38]^. The NCI-H460/R cell line is resistant to several drugs, including doxorubicin, paclitaxel, etoposide, vinblastine, and epirubicin^[37]^. Both cell lines were cultured in RPMI-1640 medium supplemented with Ultraglutamine I and 25 mM 4-(2-hydroxyethyl)-1-piperazineethanesulfonic acid (HEPES) and 10% fetal bovine serum (FBS; Biowest). The sensitive human NSCLC adenocarcinoma cell line, A549, was obtained from American Type Culture Collection (ATCC), and its MDR counterpart cell line, A549-CDR2, was established by our research group by continuous treatment with increased concentrations of paclitaxel. This cell line is resistant to paclitaxel, vinorelbine, and doxorubicin [Supplementary Figure 1] as well as to docetaxel, etoposide, and gemcitabine (unpublished data). The A549 and A549-CDR2 cells were cultured in DMEM with 4.5 g/L Glucose (Lonza; BE12-604F) and 10% FBS. To maintain the resistant phenotype of the MDR cell lines, doxorubicin and paclitaxel were added every three weeks to NCI-H460/R and A549-CDR2, respectively. For each assay, doxorubicin was added six days and paclitaxel was added three days before starting each experiment to NCI-H460/R and A549-CDR2 cells, respectively. During the performance of the Sulforhodamine B (SRB) assay, all cell lines were supplemented with 5% FBS. The non-tumorigenic breast cell line, MCF-10A (from ATCC), was cultured in DMEM/F12 Medium (Thermo Fischer Scientific), as previously described^[39]^. All cell lines were kept in a humidified incubator at 37 °C with 5% CO_2_.

### Reagents

Carboplatin (BP711), doxorubicin (D1515), paclitaxel (T7402), pentoxifylline (P1784), verapamil (v-4629), and vinorelbine (Y0000463) were obtained from Merck Life Science. Dimethyl-sulfoxide (DMSO) was used to dissolve paclitaxel and doxorubicin, whereas sterile water was used to dissolve carboplatin, pentoxifylline, and vinorelbine. Carboplatin was stored at 4 °C while the other drugs stored at -20 °C.

### Drug treatments

The NSCLC cell lines were treated with different concentrations of pentoxifylline (4, 2, 1 and 0.5 mM), H_2_O (the vehicle, control), or culture medium (blank). To study the cytotoxic effect of the combined therapies in both sensitive and MDR pairs of cell lines, as well as in the MCF-10A cell line, cells were treated with: (1) each drug individually; (2) drug combinations consisting of pentoxifylline together with paclitaxel, vinorelbine, carboplatin, or vinorelbine plus carboplatin; (3) H_2_O and/or DMSO (vehicles, control).

### Cell growth inhibition - SRB assay

SRB assay was performed according to Vichai and Kirtikara^[40]^ and as previously described^[17,41]^. The cells (at 5.0 × 10^4^ cells/mL) were plated in 96 wells and, after 24 h, treated with different drugs. After 48 h, cells were fixed with 50 µL ice-cold 10% (w/v) trichloroacetic acid, washed with distilled water, and then stained with 0.4% (w/v) SRB (Merck Life Science) in 1% (v/v) acetic acid for 30 min in the dark, and at room temperature. Cells were washed with 1% (v/v) acetic acid and then 10 mM Tris-Base was added for 5 min and absorbance measured (Synergy TM BioTek Instruments, Inc.) using Gen5TM software. Drug concentration-response curves were determined and the GI_50_ concentration (which causes 50% of cell growth inhibition) was calculated.

### Protein expression - Western Blot analysis

Cells were plated in 6-well plates (at 5.0 × 10^4^ cells/mL) for 24 h and then treated with pentoxifylline at 1 mM or with H_2_O (vehicle, control) for 48 h. Afterwards, cells were collected, centrifuged and protein extracted using Winman’s Buffer, as previously described^[17,41,42]^. After a centrifugation process, protein lysates were quantified (Bio-Rad DCTM Protein Assay Kit). Proteins (20 µg per lane) were separated on a 10% SDS-PAGE gel and then electrotransferred to a nitrocellulose membrane. The membranes were stained with Ponceau and then blocked for at least 1 h with either 5% (w/v) non-fat dry milk in Tris-buffered saline solution (TBS) with 0.1% Tween-20 (TBS-T) or 5% (w/v) bovine serum albumin (BSA) in TBS-T. Membranes were incubated overnight at 4 °C. The primary antibodies used were: anti-Pgp (sc-55510), anti-β-Catenin (sc-7963), and anti-β-Actin (sc-47778) from Santa Cruz Biotechnology; anti-CHI3L1 (AF2599) and anti-IL13Rα2 (AF146) from R&D systems; anti-ρ-ERK (9101), ERK (4695), anti-ρ-Akt (4051), Akt (9272), anti-ρ-STAT3 (9131), and anti-STAT3 (9139) from Cell Signaling. The secondary antibodies used were: anti-mouse, anti-goat, and anti-rabbit from Santa Cruz Biotechnology. The signal was detected in Amersham Hyperfilm ECL.

### Cell cycle profile analysis

The effect of pentoxifylline on the cell cycle profile was analyzed using propidium iodide (PI) staining, according to the previously described protocol^[41-43]^. After 24 h of cell seeding (5.0 × 10^4^ cells/mL), cells were exposed for 48 h to the following conditions: (1) culture medium (blank); (2) medium with H_2_O (vehicle); (3) pentoxifylline at 1 or 2 mM. After this period, cells were collected, centrifuged and the pellets fixed with cold ethanol. After at least 12 h at 4 °C, cells were centrifuged and resuspended in phosphate buffered saline (PBS) with 5 g/mL PI (Merck Life Science) and 0.1 mg/mL RNase (Invitrogen), for 30 min at 4 °C, protected from the light. All samples were analyzed by flow cytometry (BD Accuri^TM^ C6 Plus Flow Cytometer, BD Biosciences). A minimum of 15,000 events per sample were plotted after the exclusion of cell debris and aggregates. Data were analyzed using the FlowJo 10.8. Software.

### Cell death analysis

The effect of pentoxifylline on cell death was evaluated using the Annexin V-FITC Apoptosis Detection Kit (Invitrogen), as previously described^[41,42]^. After 24 h of seeding (at 5.0 × 10^4^ cells/mL), cells were treated with: (1) culture medium (blank); (2) medium with H_2_O (vehicle); or (3) pentoxifylline at 1 and 2 mM. Following 48 h of treatment, and 1 h before collecting the cells, ethanol was added to the blank condition (positive control). Cells were centrifuged and the pellet resuspended in Binding Buffer. Cell suspensions were divided into two samples: (a) auto-fluorescence; and (b) labeled samples, which were incubated with the Annexin V-FITC conjugate, followed by incubation with PI on ice. Samples were analyzed by flow cytometry (at least 15,000 events per sample were plotted, excluding cell debris and aggregates) and the percentage of dead cells was determined using the FlowJo program.

### Rhodamine-123 accumulation assay

The P-gp inhibitory activity of pentoxifylline was assessed with the Rhodamine-123 accumulation assay^[43,44]^. Cells were cultured for 24 h in 12-well plates (at 5.0 × 10^4^ cells/mL) and then treated with: (1) vehicle (H_2_O) or pentoxifylline at 1, 2, 5, or 10 mM during 1 h; (2) vehicle or pentoxifylline at 1 or 2 mM during 48 h. The sensitive cell line NCI-H460 was used as a negative control. The MDR NCI-H460/R cells under 20 µM Verapamil treatment (P-gp inhibitor) were used as a positive control. Cells were treated with or without rhodamine-123 (Merck Life Science) at 1 µM. In the end, cells were collected, centrifuged, and resuspended in cold PBS. A minimum of 15,000 events per sample were acquired (excluding cell debris and aggregates) using the Flow Cytometer.

### Association studies between *CHI3L1* gene expression and OS and drug response

Bioinformatic studies using The Cancer Genome Atlas (TCGA) database (https://www.cancer.gov/tcga) were performed to assess the possible association between *CHI3L1* gene expression and OS of 994 patients with NSCLC. Moreover, possible associations between *CHI3L1* gene expression and OS of NSCLC patients under different drug treatments (carboplatin, vinorelbine and carboplatin plus vinorelbine) were analyzed.

### Determination of the fold change of the combined therapies vs. conventional chemotherapies

The fold change was determined for each drug treatment and in each pair of sensitive and MDR cell lines (NCI-H460 *vs.* NCI-H460/R and A549 *vs.* A549-CDR2), at the concentrations used for the drug combinations and using the following formula: % of Cell growth inhibition of the combined treatment of a chemotherapeutic drug with pentoxifylline/% of Cell growth inhibition of the conventional chemotherapeutic drug.

### Statistical analysis

The two-tailed unpaired *t*-test was applied using the GraphPad Prism 8.0 software. Results represent the mean ± standard error of the mean (SEM), and a minimum of three independent experiments were performed. Statistical significance was considered whenever *P* ≤ 0.05. Non-statistical significance (ns) whenever *P* > 0.05.

## RESULTS

### Pentoxifylline decreased the growth of sensitive and MDR counterpart NSCLC cell lines

The effect of pentoxifylline (48 h treatment) on NSCLC cell growth was assessed using the SRB assay, and the GI_50_ concentration calculated based on the determination of the drug concentration-response curves [Supplementary Figure 2]. Table 1 demonstrates that pentoxifylline, at concentrations below 2 mM, reduced the growth of all the cell lines. Moreover, pentoxifylline had a slightly higher effect in the drug-sensitive cell lines (NCI-H460 and A549), compared to their counterpart MDR cell lines (NCI-H460/R and A549-CDR2).

**Table 1:** GI_50_ concentrations of pentoxifylline in two pairs of sensitive and MDR counterpart NSCLC cell lines

**Drug**	GI_50_^*^ (mM)			
	**NCI-H460**	**NCI-H460/R**	**A549**	**A549-CDR2**
Pentoxifylline	0.96 ± 0.08	1.17 ± 0.17	1.23 ± 0.07	1.53 ± 0.04

^*^GI_50_ concentration (which causes 50% inhibition of cell growth) after 48 h of drug treatment (determined with the SRB assay). MDR: Multidrug resistance; NSCLC: non-small cell lung cancer; SRB: sulforhodamine B.

### Pentoxifylline decreased the expression levels of P-gp, CHI3L1 and STAT3 in NSCLC cell lines

**Figure 1:**
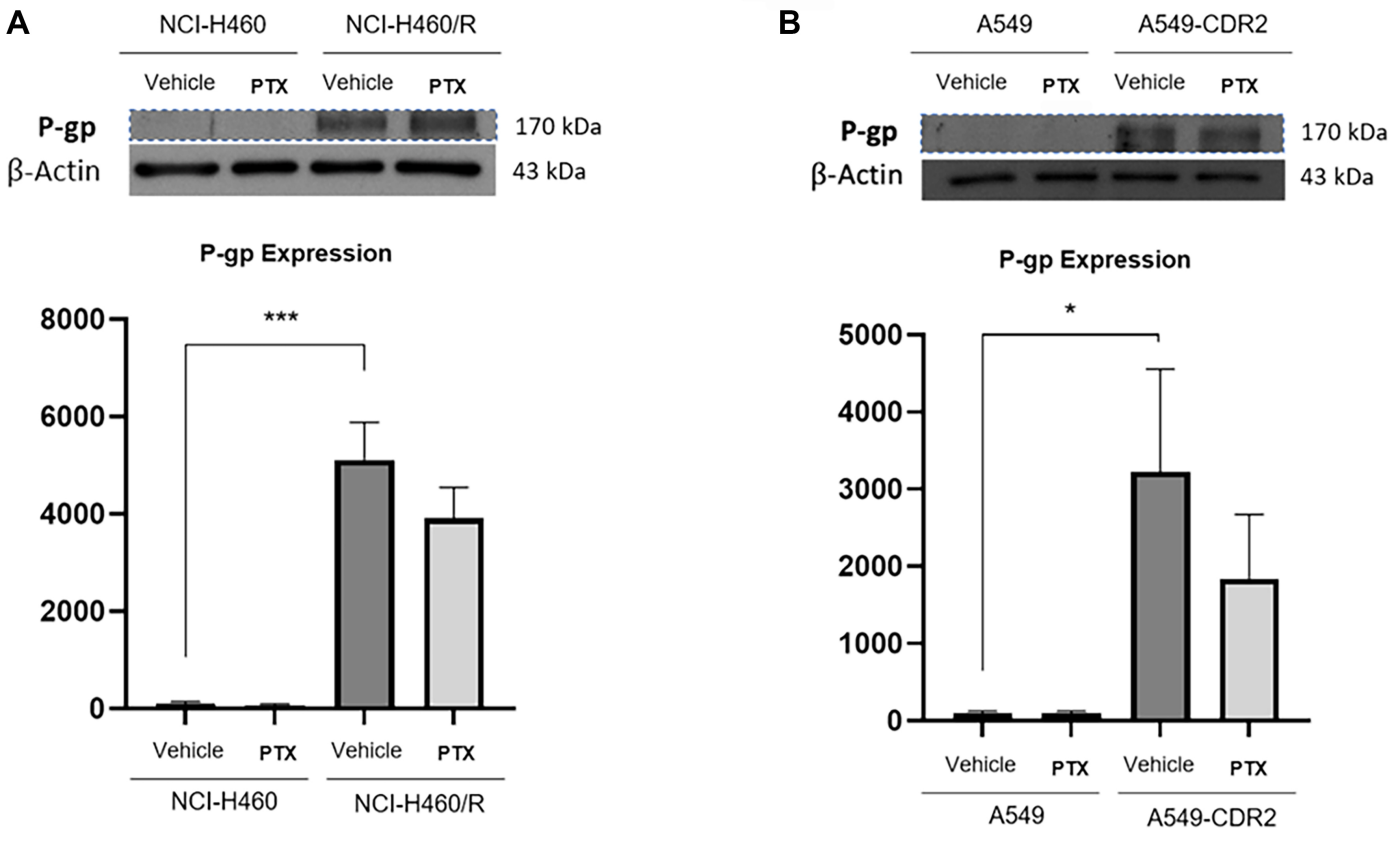
Effect of pentoxifylline on the expression levels of P-gp, determined by Western Blot, on (A) NCI-H460 *vs.* NCI-H460/R and (B) A549 *vs.* A549-CDR2 cell lines, treated with vehicle (H_2_O) or pentoxifylline at 1.0 mM after 48 h. β-Actin was used as a loading control. Data are presented as a percentage of expression. ^*^P ≤ 0.05; ^***^P < 0.001. P-gp: P-glycoprotein.

To unravel the mechanism of action of pentoxifylline, the effect of this drug (at 1 mM for 48 h) on the expression levels of P-gp (MDR-related protein), CHI3L1 (known target of pentoxifylline) and its main downstream proteins, such as β-catenin, phospho(p)-Akt, phospho(p)-ERK, and phospho(p)-STAT3 was studied. As expected, the results obtained [Figure 1] confirmed the higher expression levels of P-gp in the NCI-H460/R cell line, compared to NCI-H460 cells^[45,46]^. The same trend was found in the A549/A549-CDR cell lines. Pentoxifylline slightly decreased P-gp expression, although not significantly, in both resistant cell lines.

**Figure 2:**
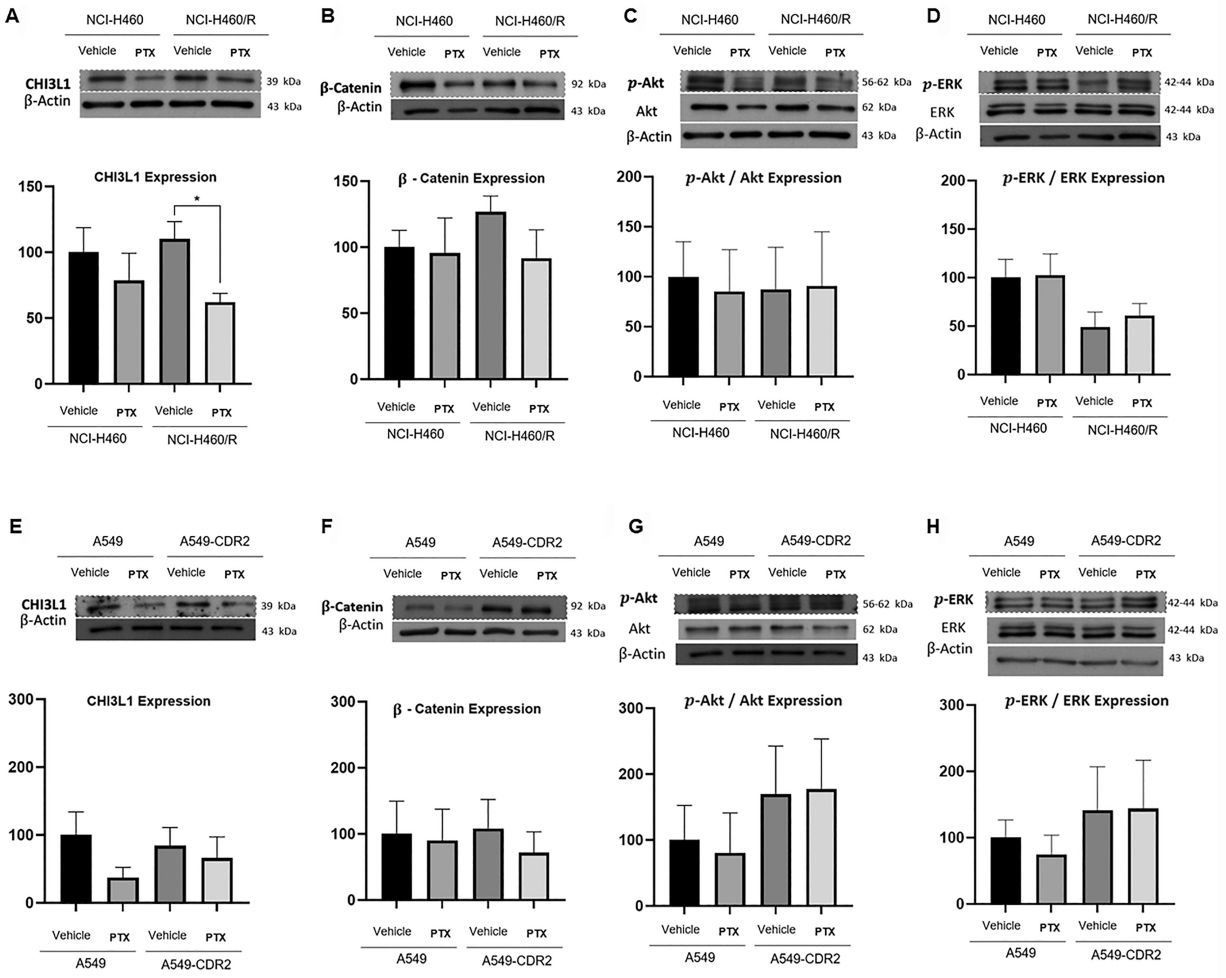
Effect of 48-h treatment with 1 mM pentoxifylline on the expression levels of CHI3L1 and its downstream proteins (β-Catenin, p-Akt and p-ERK), determined by Western Blot on (A-D) NCI-H460 *vs.* NCI-H460/R and on (E-H) A549 *vs.* A549-CDR2 cell lines. The effect of the vehicle (H_2_O) was also analyzed. β-Actin was used as a loading control. Data are presented as a percentage of expression. ^*^P ≤ 0.05. CHI3L1: Chitinase 3-like-1; ERK: extracellular signal-regulated kinase.

Results also showed that pentoxifylline significantly decreased CHI3L1 expression in the NCI-H460/R cells [Figure 2] and slightly reduced CHI3L1 expression in both sensitive cell lines. Pentoxifylline did not affect the expression levels of the CHI3L1 receptor, IL-13Rα2 [Supplementary Figure 3]. Additionally, a slight decrease in the expression levels of β-catenin was observed in both MDR cell lines treated with pentoxifylline. No effect of pentoxifylline was observed on the activation levels (expression of phospho/total) of ERK and Akt in any of the NSCLC cell lines.

**Figure 3:**
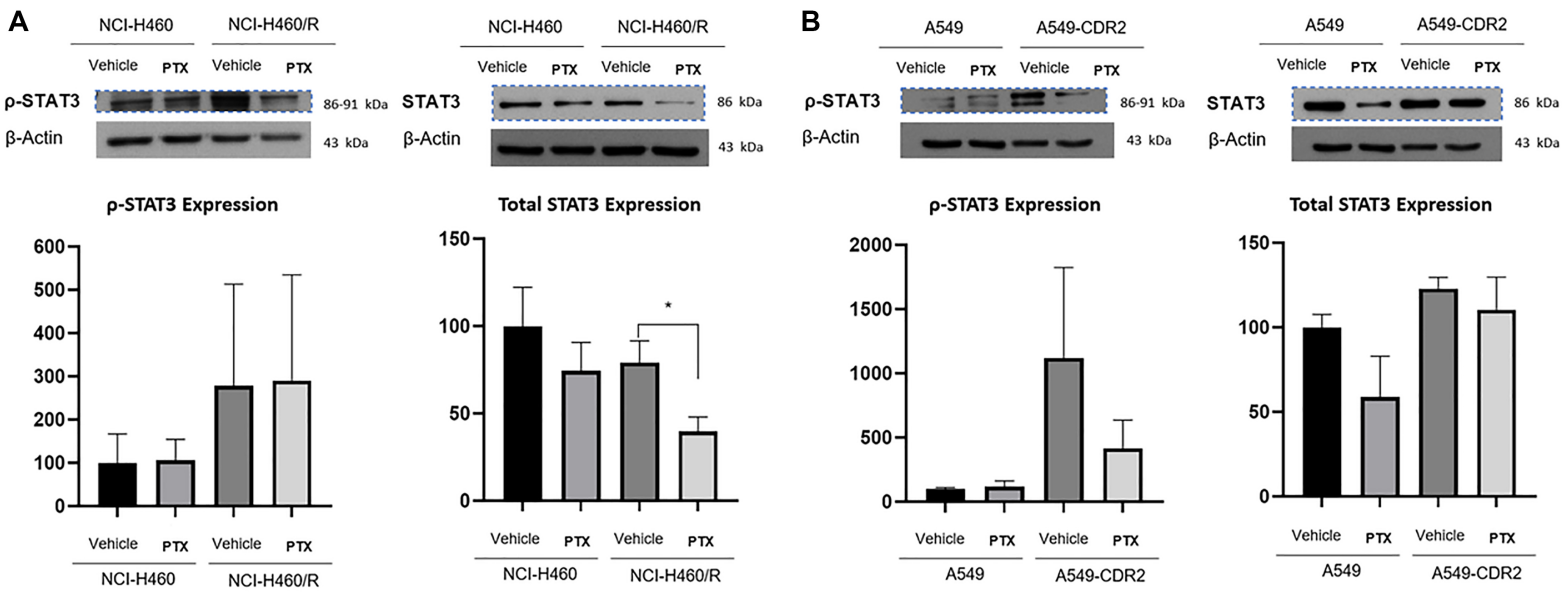
Effect of 48-h treatment with 1 mM pentoxifylline on the expression levels of total and phosphorylated STAT3, determined by Western Blot on (A) NCI-H460 *vs.* NCI-H460/R and (B) A549 *vs.* A549-CDR2 cell lines. The effect of the vehicle (H_2_O) was also analyzed. β-Actin was used as a loading control. Data are presented as a percentage of expression. ^*^P ≤ 0.05. STAT3: signal transducer and activator of transcription 3.

Moreover, pentoxifylline significantly decreased the total STAT3 expression levels in the resistant NCI-H460/R cell line. In the A549-CDR2 cell line, pentoxifylline decreased STAT3 phosphorylation (p-STAT3) levels, although not significantly [Figure 3].

### Pentoxifylline caused an increase in the percentage of cells in the G0/G1 phase of the cell cycle

**Figure 4:**
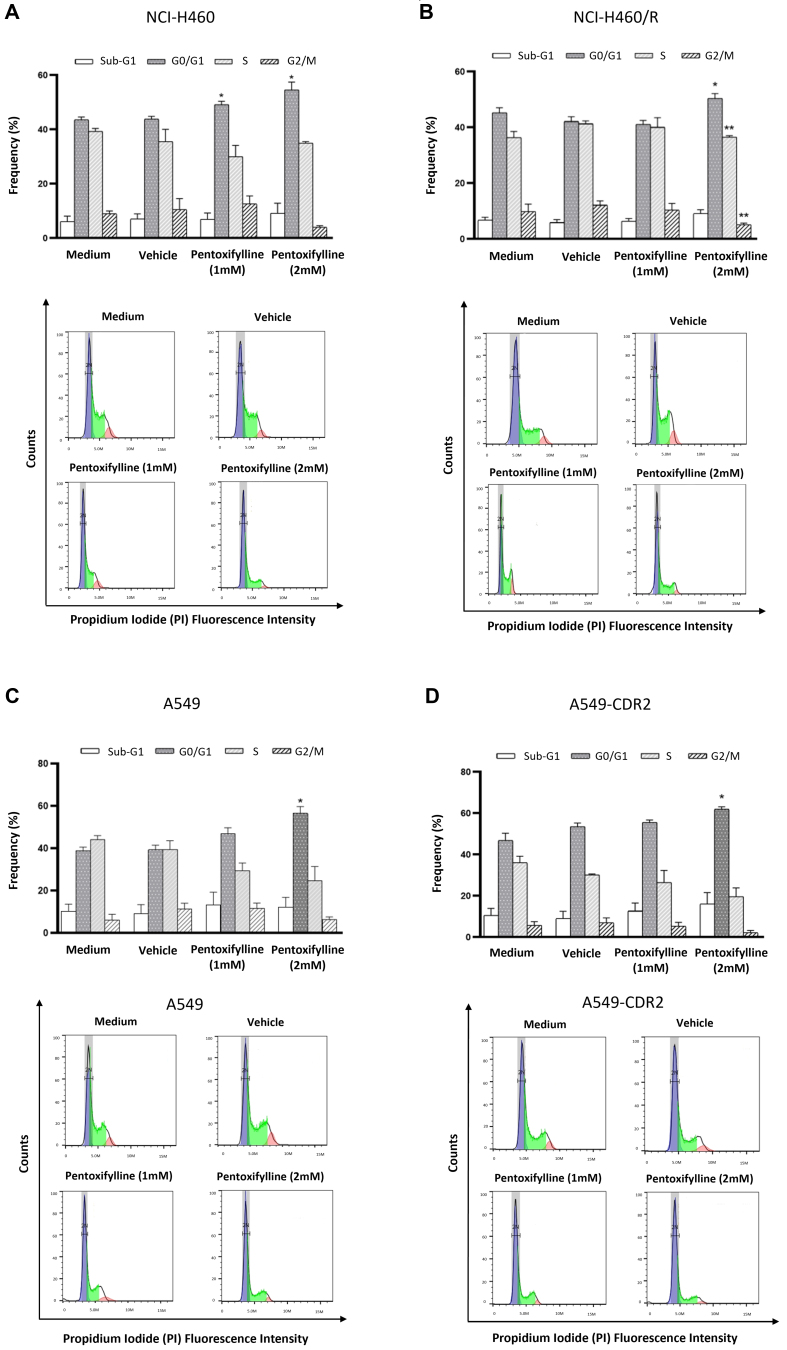
Effect of 1 or 2 mM pentoxifylline 48 h treatment on the cell cycle profile of (A) NCI-H460 *vs.* (B) NCI-H460/R and (C) A549 *vs.* (D) A549-CDR2 cell lines, analyzed by flow cytometry. The frequency of the cell cycle phases (top panels), as well as the representative cell cycle histograms (bottom panels), are presented. ^*^P ≤ 0.05; ^**^P < 0.01.

The effect of pentoxifylline on the cell cycle profile was assessed. As shown in Figure 4, pentoxifylline significantly increased the percentage of cells in the G0/G1 phase of the cell cycle in NCI-H460 and NCI-H460/R cell lines. In the NCI-H460/R cell line, a significant decrease in the percentage of cells in the S and G2/M phases was also observed. Consistently, pentoxifylline, at the higher concentration tested, significantly increased the percentage of cells in the G0/G1 phase in A549 and A549-CDR2 cell lines. Moreover, pentoxifylline slightly increased the percentage of cells in the sub-G1 phase (indicative of apoptosis) in all the cell lines.

### Pentoxifylline increased cell death of the sensitive NCI-H460 NSCLC cell line

**Figure 5:**
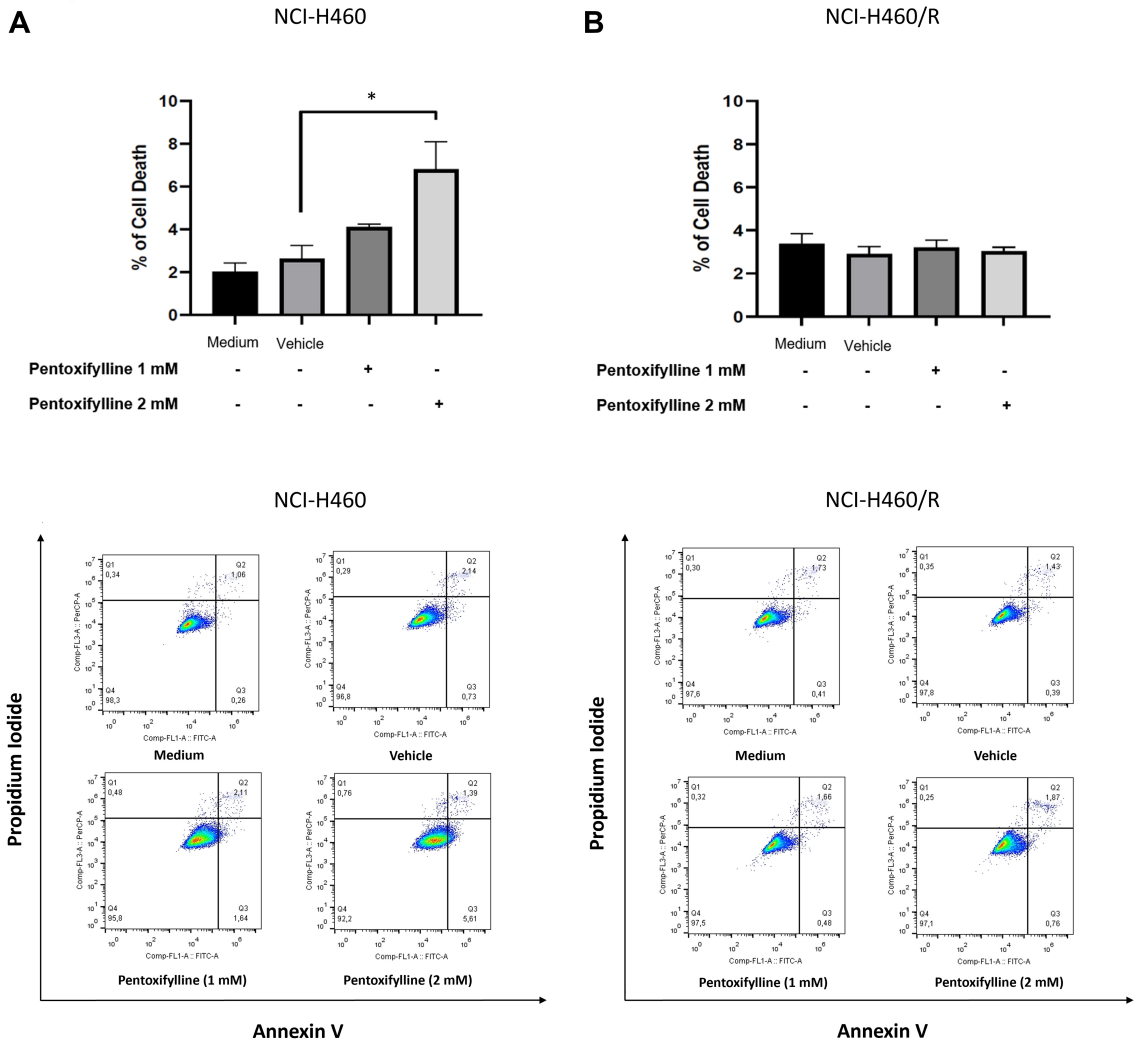
Effect of 48 h treatment with 1 or 2 mM pentoxifylline on the percentage of cell death of (A) sensitive NCI-H460 and (B) MDR NCI-H460/R NSCLC cells, determined by flow cytometry. The effect of the vehicle at the highest concentration was also tested. The frequency of dead cells for each condition (top panels) and the representative dot plot histograms (bottom panels) are shown. ^*^P ≤ 0.05. MDR: Multidrug resistance; NSCLC: non-small cell lung cancer.

The effect of pentoxifylline on cell death was evaluated. Results demonstrated that pentoxifylline significantly increased the percentage of cell death in the NCI-H460 cells in a concentration-dependent manner [Figure 5]. This effect, however, was not observed in the resistant NCI-H460/R cells.

### Pentoxifylline did not affect P-gp activity

**Figure 6:**
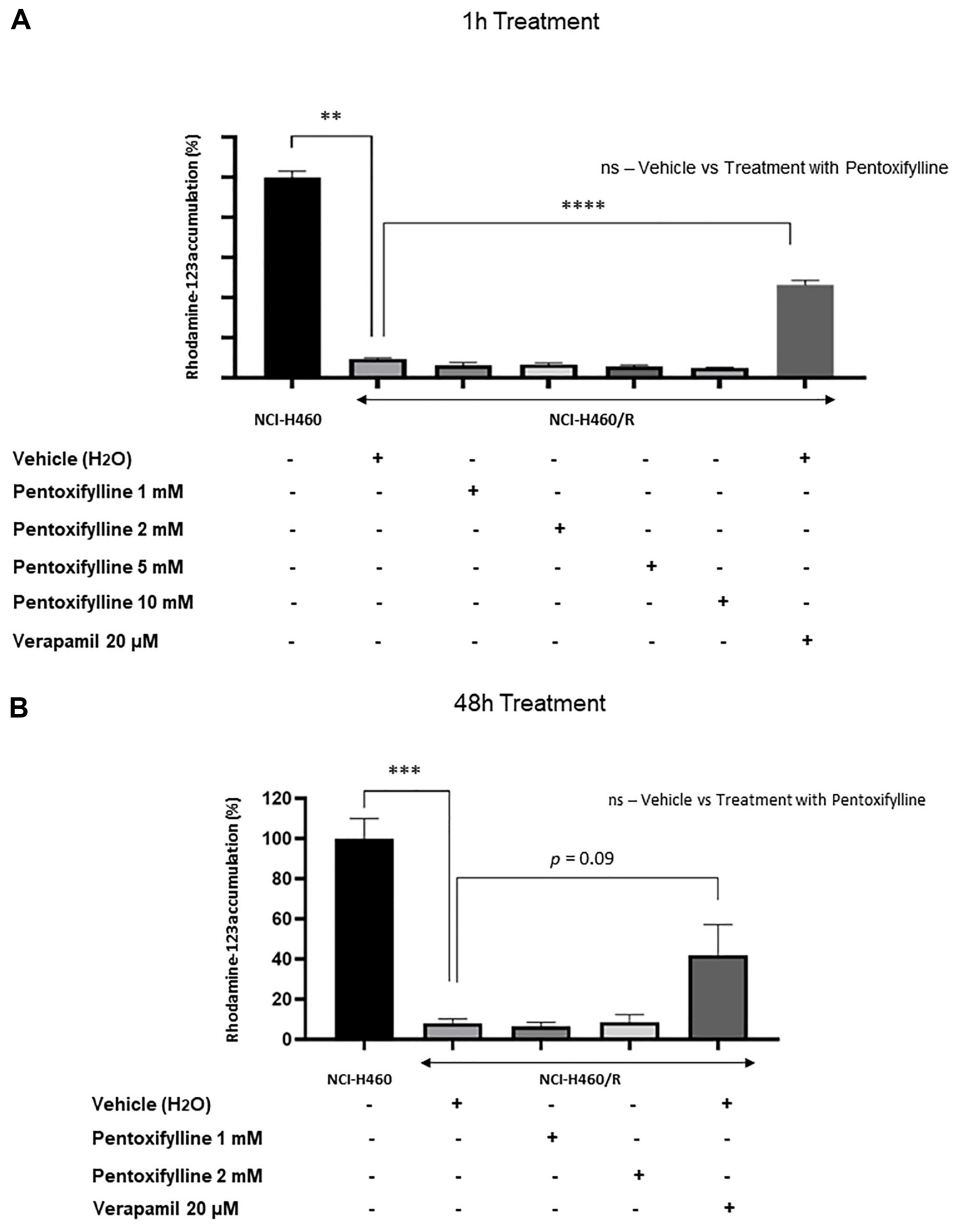
Effect of (A) 1-h and (B) 48-h treatments with different concentrations of pentoxifylline on P-gp activity of the NCI-H460/R cells, determined by the Rhodamine-123 accumulation assay. NCI-H460 cells were used as a negative control. The positive control consisted of NCI-H460/R cells treated with 20 μM verapamil. ^**^P < 0.01; ^***^P < 0.001; ^****^P < 0.0001. P-gp: P-glycoprotein.

Previous data revealed that pentoxifylline reduced the expression levels of the drug efflux pump P-gp in both MDR NSCLC cell lines. As shown in Figure 6, pentoxifylline did not cause alteration in P-gp activity following 1 or 48 h drug treatment, as there was no increase in the amount of intracellular rhodamine-123 accumulated on NCI-H460/R cells under drug treatment.

### *CHI3L1* gene expression was associated with the low response of NSCLC patients to vinorelbine treatment

**Figure 7:**
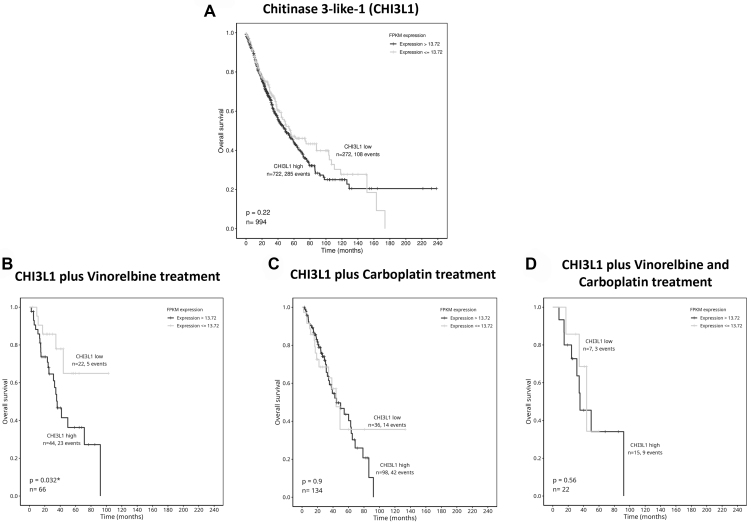
OS and *CHI3L1* gene expression in 994 NSCLC cases, using Kaplan-Meier curves. Comparison between low and high expression of CHI3L1 in (A) NSCLC patients and (B) under vinorelbine, (C) carboplatin, or (D) vinorelbine plus carboplatin treatments. OS: Overall survival; CHI3L1: chitinase 3-like-1; NSCLC: non-small cell lung cancer.

The association of *CHI3L1* gene expression with the OS of 994 patients with NSCLC, and the response of those patients to different chemotherapeutic regimens was studied using the TCGA database. Figure 7 demonstrates that high expression of CHI3L1 is not associated with poor OS of NSCLC patients. Importantly, NSCLC patients under vinorelbine treatment, and with high expression levels of CHI3L1, do not benefit from this therapy. The same tendency is observed when NSCLC patients are under carboplatin treatment, although in this case, the difference is not statistically significant. In NSCLC patients treated with carboplatin plus vinorelbine, the expression of CHI3L1 does not affect the response to the drug treatment.

### Pentoxifylline sensitized all NSCLC cell lines to different drug regimens

**Figure 8:**
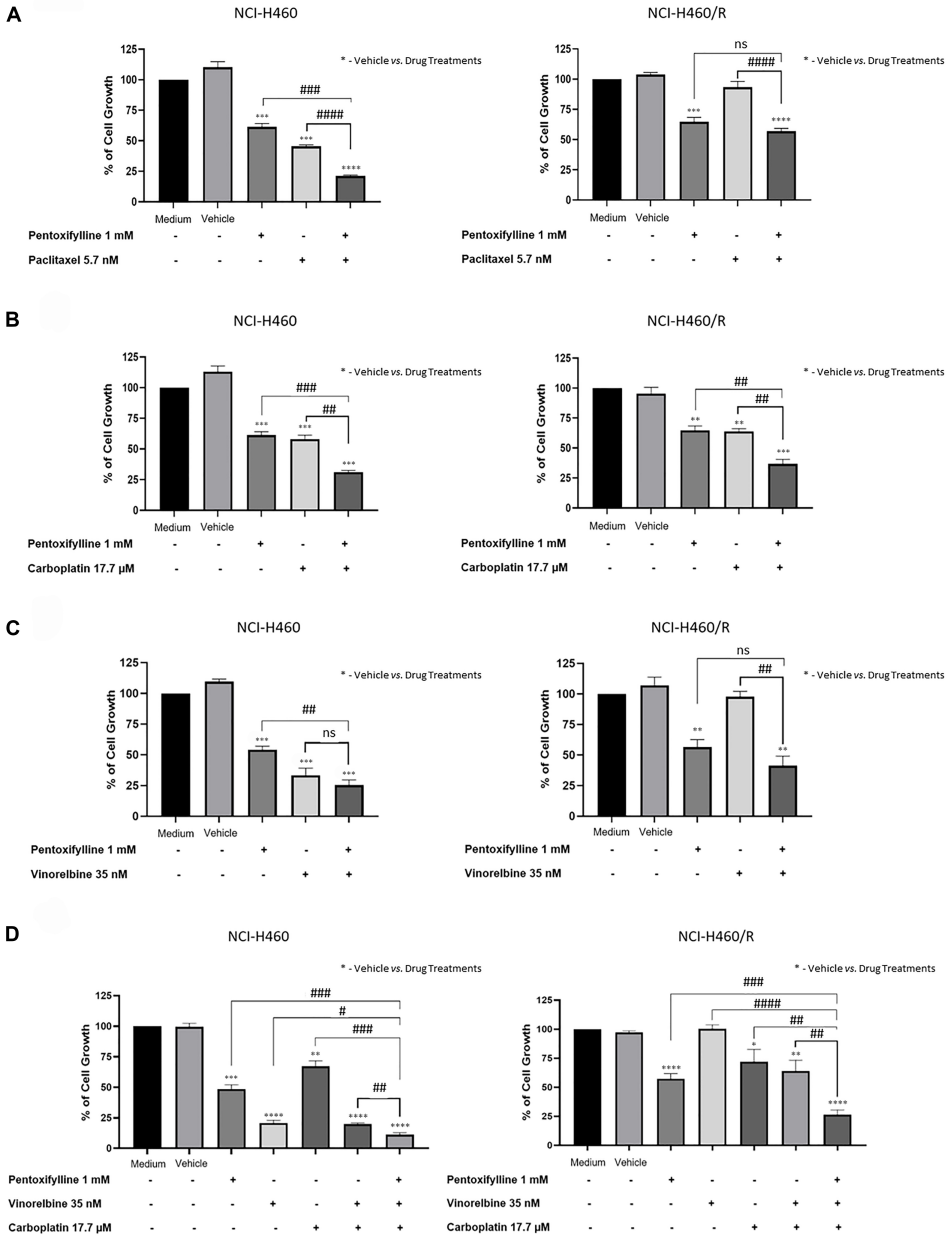
Effect of pentoxifylline in combination with different anticancer drugs on the percentage of cell growth of NCI-H460 and NCI-H460/R cell lines, assessed by the SRB assay. Cells were treated for 48 h with pentoxifylline at 1.0 mM and (A) paclitaxel at 5.7 nM, (B) carboplatin at 17.7 µM, (C) vinorelbine at 35 nM, and (D) vinorelbine at 35 nM plus carboplatin at 17.7 μM. The effect of the vehicle at the highest concentration was also tested. Data are presented as a percentage of cell growth. ^*,#^*P* ≤ 0.05; ^**,##^*P* < 0.01; ^***,###^*P* < 0.001; ^****,####^*P* < 0.0001; ns: *P* > 0.05. SRB: Sulforhodamine B.

The combination treatment consisting of pentoxifylline with different drug regimens, namely paclitaxel, vinorelbine, carboplatin, and vinorelbine plus carboplatin, was evaluated in sensitive and MDR NSCLC cell lines using the SRB assay. The results [Figure 8] showed that pentoxifylline in combination with paclitaxel significantly decreased the percentage of cell growth in the sensitive NCI-H460 cell line compared with pentoxifylline or paclitaxel alone, and in the resistant NCI-H460/R cell line but only compared to paclitaxel alone. Regarding the combined treatment consisting of pentoxifylline with carboplatin, a significant decrease in the percentage of cell growth was observed in both sensitive cell lines and in the resistant NCI-H460/R cell line, compared to treatment with pentoxifylline or carboplatin alone. When pentoxifylline was combined with vinorelbine, a slight reduction in the percentage of NCI-H460 cell growth, and a significant effect on the percentage of NCI-H460/R cell growth, were observed compared with vinorelbine alone. Finally, both NCI-H460 and NCI-H460/R cell lines responded significantly better to treatment consisting of pentoxifylline in combination with vinorelbine plus carboplatin, compared with these drugs individually.

**Figure 9:**
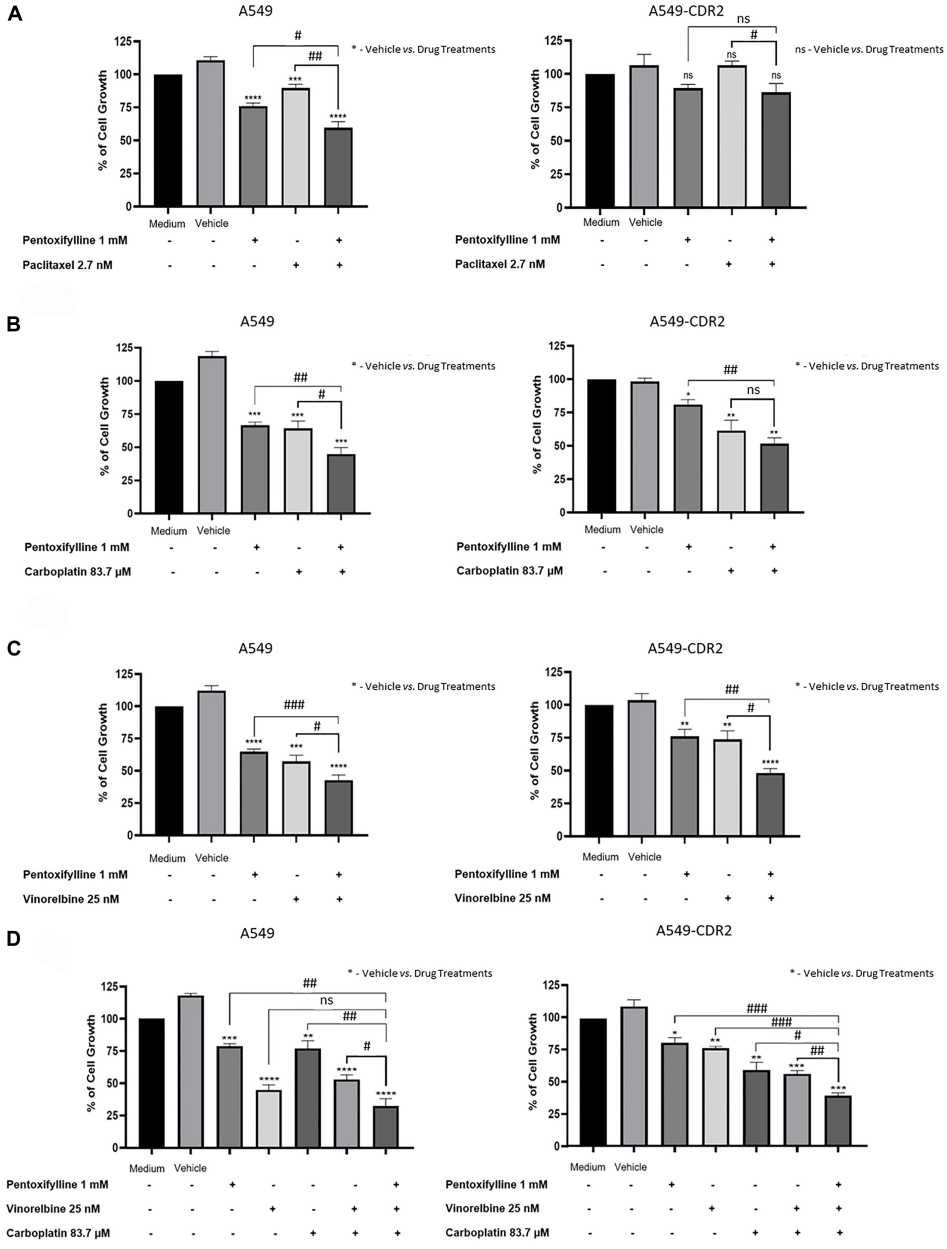
Effect of pentoxifylline in combination with different anticancer drugs on the percentage of cell growth of A549 and A549-CDR2 cell lines, assessed by the SRB assay. Cells were treated for 48 h with pentoxifylline at 1.0 mM and (A) paclitaxel at 2.7 nM, (B) carboplatin at 83.7 μM, (C) vinorelbine at 25 nM, or (D) vinorelbine at 25 nM plus carboplatin at 83.7 μM. Data are presented as a percentage of cell growth. ^*,#^*P* ≤ 0.05; ^**,##^*P* < 0.01; ^***,###^*P* < 0.001; ^****,####^*P* < 0.0001; ns: *P* > 0.05.

Further, the chemosensitizing effect of pentoxifylline was validated in another pair of sensitive/MDR NSCLC cell lines, A549/A549-CDR2. The results [Figure 9] demonstrated that pentoxifylline combined with paclitaxel significantly decreased the percentage of cell growth in both cell lines, compared with paclitaxel alone. However, compared with pentoxifylline alone, the effect was only significant in the sensitive cell line. Moreover, pentoxifylline combined with carboplatin significantly decreased the percentage of cell growth in A549 cells, compared with carboplatin alone or with pentoxifylline alone. In A549-CDR2 cells, the effect was only significant compared with pentoxifylline alone. Additionally, pentoxifylline, in combination with vinorelbine, significantly decreased the percentage of cell growth in both A549 and A549-CDR2 cells. Finally, the inhibition of cell growth exhibited a significantly greater magnitude when pentoxifylline was combined with vinorelbine plus carboplatin, in both sensitive and MDR cells, as opposed to when vinorelbine plus carboplatin was used. However, in the sensitive cells, the effect of the triple drug combination was not significantly different from the effect of treatment with vinorelbine alone.

Importantly, the fold change of the percentage of cell growth inhibition of the different combination therapies (conventional chemotherapy with pentoxifylline) *vs.* conventional chemotherapy, for both pairs of NSCLC cell lines, was calculated [Table 2]. Values above 1 mean that the combination of a particular chemotherapeutic drug with pentoxifylline was more effective in decreasing cell growth than the chemotherapeutic drug alone. Thus, higher fold changes suggest more potent combination treatments. Table 2 shows that pentoxifylline combined with the different chemotherapeutic drugs decreased the percentage of cell growth more efficiently than the chemotherapeutic drugs alone. Interestingly, in the NCI-H460/R cell line, pentoxifylline in combination with vinorelbine and with vinorelbine plus carboplatin have the highest fold changes (values above 2.0).

**Table 2:** Fold change of the percentage of growth inhibition of the NSCLC cell lines following treatment with chemotherapeutic drugs combined with pentoxifylline *vs.* without pentoxifylline

**NSCLC cell lines**	**Combination treatment consisting of pentoxifylline with**			
	**Paclitaxel**	**Vinorelbine**	**Carboplatin**	**Vinorelbine plus carboplatin**
NCI-H460	2.14	1.33	1.86	1.78
NCI-H460/R	1.64	2.36	1.74	2.42
A549	1.5	1.25	1.41	1.62
A549-CDR2	1.23	1.53	1.19	1.43

NSCLC: Non-small cell lung cancer.

The cytotoxic effect of pentoxifylline combined with conventional chemotherapeutic drugs (paclitaxel, vinorelbine, carboplatin, and vinorelbine plus carboplatin) was also evaluated in the MCF-10A cells using the SRB assay. Results [Supplementary Figure 4] demonstrated that, at least at the concentrations tested, none of the combination treatments caused a significant increase in cytotoxicity, compared to the conventional therapy alone.

## DISCUSSION

MDR is commonly observed in patients with NSCLC treated with different drug regimens currently applied in the clinical practice^[7]^, and one of the causes has been associated with the presence of a highly resistant fibrotic stroma^[12]^. Thus, combining an anti-fibrotic drug with conventional chemotherapeutic drugs may improve therapeutic response of NSCLC cells.

Recently, our research group demonstrated that pentoxifylline, an approved drug for the treatment of vascular diseases with anti-fibrotic properties, sensitized pancreatic cancer cells to gemcitabine treatment^[17]^. Importantly, pentoxifylline reversed drug resistance induced by CHI3L1 in pancreatic cancer cells^[17]^. In fact, pentoxifylline is described as a CHI3L1 inhibitor, and overexpression of CHI3L1 has been associated with poor survival in different types of cancer^[47]^. Therefore, this work aimed to evaluate the effect of pentoxifylline on sensitive and MDR NSCLC cells and disclose its mechanism of action, as well as to verify the sensitizing effect of pentoxifylline to different chemotherapeutic regimens.

This work revealed that pentoxifylline reduced the growth of two pairs of sensitive and MDR NSCLC cell lines at concentrations below 2 mM. The mechanism of action of pentoxifylline was then explored in NSCLC cells. Pentoxifylline reduced the expression levels of P-gp but did not affect its activity. Barancik *et al.* also showed that pentoxifylline reduced P-gp expression in a MDR mouse leukemia cell line^[48]^. Moreover, Drobná *et al.* showed that pentoxifylline decreased the efflux activity of P-gp in vincristine-resistant mouse leukemic cells^[49]^. Importantly, the present work also showed that pentoxifylline decreased the expression levels of CHI3L1 in the pairs of sensitive and MDR NSCLC cells under study, without affecting the expression levels of some of the reported CHI3L1 downstream proteins, such as Akt and Erk. A slight decrease in β-catenin expression levels (another CHI3L1 downstream protein) was observed. These results suggest that the observed effect of pentoxifylline might be achieved by decreasing CHI3L1 expression levels. Indeed, it has been reported that pentoxifylline downregulates CHI3L1^[27,50]^. Contrary to our data, other authors showed the ability of pentoxifylline (when tested at higher concentrations than in this work and in a triple-negative breast cancer cell line) to downregulate the Akt and ERK signaling pathways^[23]^.

Additionally, this work demonstrated that pentoxifylline decreased STAT3 expression levels in one of the MDR cell lines, which corroborates previous studies demonstrating that pentoxifylline impairs cancer cells’ ability to migrate through the downregulation of STAT3 signaling in B16-F10 murine melanoma^[51]^ and in A375 human melanoma cells^[52]^. Interestingly, STAT3 is also a downstream signaling protein to CHI3L1^[53]^. Although the exact mechanism of STAT3 activation by CHI3L1 is not fully understood, one possibility is that CHI3L1 activates STAT3 through receptor interaction^[36]^. In fact, Lee *et al.* reported that CHI3L1 knockdown decreased STAT3 activity, which in turn decreased cancer cell growth in lung cancer^[54]^. Moreover, there are some studies that demonstrate a positive feedback loop between CHI3L1 and STAT3, since STAT3 also mediates the transcription of the *CHI3L1* gene^[55,56]^. Interestingly, the results presented in the present study demonstrated that pentoxifylline significantly decreased both CHI3L1 and STAT3 expression levels in the NCI-H460/R cell line. In the other NSCLC cell lines, a slight decrease in CHI3L1 and STAT3 expression levels was also observed. Altogether, our results suggest that pentoxifylline might also exert its effect on NSCLC through inhibition of the CHI3L1/STAT3 signaling.

Pentoxifylline was able to interfere with the cell cycle profile of both sensitive and MDR NSCLC cells by increasing the percentage of cells in the G0/G1 phase. Moreover, this drug caused cell death in the sensitive NCI-H460 cells. Other studies have also reported the effect of pentoxifylline on the cell cycle profile and cell death in other cancer types. For instance, Castellanos-Esparza *et al.*^[57]^ and Wang *et al.*^[25]^ demonstrated that pentoxifylline caused cell cycle arrest in G0/G1 phase and increased cell death, in a triple-negative breast cancer cell line and in hepatocellular carcinoma cells, respectively. In the present work, pentoxifylline caused apoptosis in the NCI-H460 cells but not in the resistant counterpart cells. The explanation for this difference is unknown but may be due to the presence of gene mutations responsible for escape from apoptosis in the MDR cell line^[38]^.

The TCGA data analysis revealed that high expression of *CHI3L1* gene is not associated with low OS of NSCLC patients, even though several studies have reported that high serum levels of CHI3L1 are associated with low OS in patients with several types of cancers^[33,34,47,58]^. Notably, by analyzing this database, NSCLC patients with high expression levels of CHI3L1 and treated with vinorelbine have a worse OS than NSCLC patients with low CHI3L1 expression levels. These findings propose that high *CHI3L1* gene expression is associated with lower response to vinorelbine in NSCLC patients. Thus, combining conventional chemotherapy with CHI3L1 inhibitors may improve the therapeutic outcome of NSCLC patients. In line with this data, Ying-Cheng Chiang *et al.*^[59]^ and Xavier *et al.*^[17]^ demonstrated that CHI3L1 overexpression negatively affects the response of epithelial ovarian carcinoma cells to paclitaxel and pancreatic cancer cells to gemcitabine treatment, respectively.

Interestingly, the data presented in this study revealed that pentoxifylline sensitized NCI-H460 cells to paclitaxel treatment, and both NCI-H460 and NCI-H460/R cells to carboplatin and to vinorelbine plus carboplatin treatments. Moreover, pentoxifylline sensitized the A549 cell line to paclitaxel, vinorelbine, and carboplatin treatments, and sensitized the A549-CDR2 cell line to vinorelbine and to vinorelbine plus carboplatin treatments. Thus, the combination of pentoxifylline with some chemotherapeutic drugs increased the inhibition of cell growth, compared to the individual treatments, without presenting increased cytotoxicity to non-tumorigenic cells. Interestingly, Ohsaki *et al.* also demonstrated the benefit of combining pentoxifylline with conventional chemotherapy, namely cisplatin and etoposide, in other NSCLC cell lines, PC-9 and PC-14^[22]^.

Altogether, this work demonstrated the advantage of combining pentoxifylline with different conventional drug regimens in both sensitive and MDR NSCLC cell lines. Nevertheless, we are aware that this study has some limitations. For instance, different mechanisms of action of pentoxifylline were detected in the different NSCLC cell lines studied, particularly regarding the effect of pentoxifylline on cell death and on the expression levels of CHI3L1 and its downstream proteins. These may be due to the different histological features of both counterpart pairs of NSCLC cell lines tested, together with differences between sensitive and resistant phenotypes. In the future, it would be interesting to further understand the effect of pentoxifylline in cells with different genetic backgrounds. Another point to consider is that A549 and NCI-H460 cell lines have the Nfr2-Keap1 antioxidant response pathway activated^[60]^. Several studies demonstrated that overactivation of this pathway supports cancer progression and protects cancer cells from oxidative damage, leading to chemoresistance^[61,62]^. In fact, it is known that ERK and Akt signaling pathways upregulate Nrf2 and thus increase the Nrf2-Keap1 signaling^[62]^. Since ERK and Akt pathways are downstream of CHI3L1, therapies targeting CHI3L1 may be beneficial in downregulating the Nrf2-Keap1 pathway. Although this work was not able to observe a significant decrease in phospho-Akt and phospho-ERK levels following pentoxifylline treatment, other studies reported that CHI3L1 inhibition led to decreased activity in these pathways^[34]^, and thus may have an impact on the Nfr2-Keap1 pathway. On the other hand, a recent publication reports that pentoxifylline can increase Nrf2 in non-cancer cells^[63]^. However, in the context of tumor cells, the effect of pentoxifylline on the Nfr2-Keap1 pathway is not yet known and therefore more studies are needed. Finally, another important issue is that the effect of the drug combinations should be exploited in more advanced cell models, better resembling tumors and their microenvironment, such as in 3D advanced cell-based models (spheroids and/or patient-derived tumoroids) co-cultured with cells from the tumor microenvironment (TME). This is particularly important since CHI3L1 has been found overexpressed both in tumor cells and in cells from the TME, namely tumor-associated macrophages and cancer-associated fibroblasts. This would enable us to verify whether pentoxifylline can also counteract the effect of CHI3L1 released by these TME cells.

In conclusion, this study provides insights into the possibility of combining pentoxifylline with different chemotherapeutic drug regimens to improve the therapeutic response of NSCLC patients, even in cases of MDR. Moreover, the presented data reinforce the potential of CHI3L1 as a promising therapeutic target in NSCLC.

## DECLARATIONS

### Availability of data and materials

All data supporting our findings are available from the corresponding author upon request.

### Copyright

© The Author(s) 2024.
