# Supplementary material for: Chemosensitizing effect of pentoxifylline in sensitive and multidrug-resistant non-small cell lung cancer cells
Source: Cancer Drug Resist. 2024 May 20;7:19. doi: 10.20517/cdr.2024.04 (PMC11149106; doi:10.20517/cdr.2024.04)
Supplement: Supplementary file 1 [file cdr-7-19-SupplementaryMaterials.pdf]

Matos *et al. Cancer Drug Resist* 2024;7:19
DOI: 10.20517/cdr.2024.04

Cancer Drug Resistance

Supplementary Materials

Chemosensitizing effect of pentoxifylline in sensitive and multidrug-resistant non-small cell lung cancer cells

Beatriz S. Matos[1,2], Sara Peixoto da Silva[1,2,3], M. Helena Vasconcelos[1,2,3], Cristina P. R. Xavier[1,2,4,5]

[1]i3S - Instituto de Investigação e Inovação em Saúde, Universidade do Porto, Rua Alfredo Allen 208, Porto 4200-135, Portugal.
[2]Cancer Drug Resistance Group, IPATIMUP - Institute of Molecular Pathology and Immunology of the University of Porto, Rua Alfredo Allen 208, Porto 4200-135, Portugal.
[3]Department of Biological Sciences, FFUP - Faculty of Pharmacy, University of Porto, Rua de Jorge Viterbo Ferreira 228, Porto 4050-313, Portugal.
[4]UCIBIO - Applied Molecular Biosciences Unit, Toxicologic Pathology Research Laboratory, University Institute of Health Sciences (1H-TOXRUN, IUCS-CESPU), Gandra 4585-116, Portugal.
[5]Associate Laboratory i4HB - Institute for Health and Bioeconomy, University Institute of Health Sciences - CESPU, Gandra 4585-116, Portugal.

Correspondence to: Prof. M. Helena Vasconcelos, Dr. Cristina P. R. Xavier, i3S - Instituto de Investigação e Inovação em Saúde, Universidade do Porto, Rua Alfredo Allen 208, Porto 4200-135, Portugal. E-mail: hvasconcelos@ipatimup.pt; cristinax@ipatimup.pt

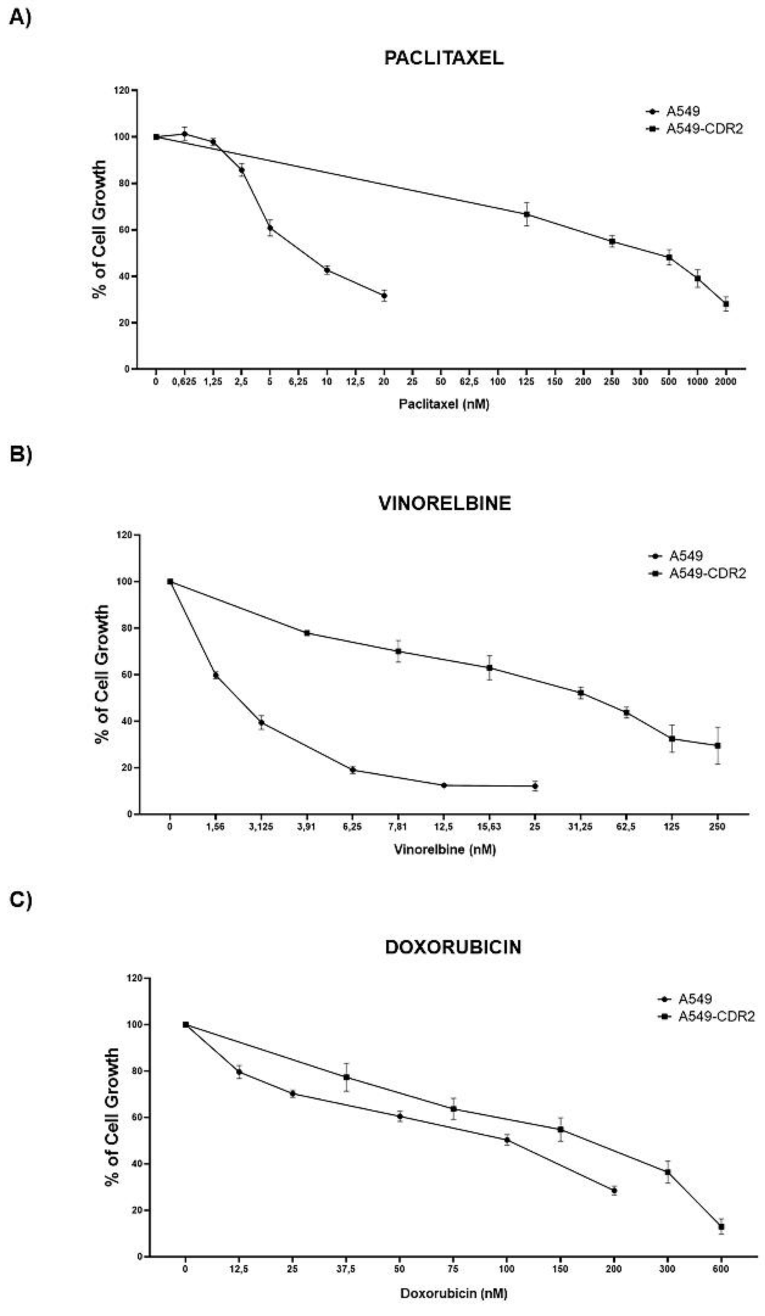

Supplementary Figure 1. Concentration-response curves of (A) paclitaxel, (B) vinorelbine and (C) doxorubicin on A549 *vs.* A549-CDR2 cell lines. Data is presented as percentage of cell growth and are the mean ± SEM of a minimum of three independent experiments.

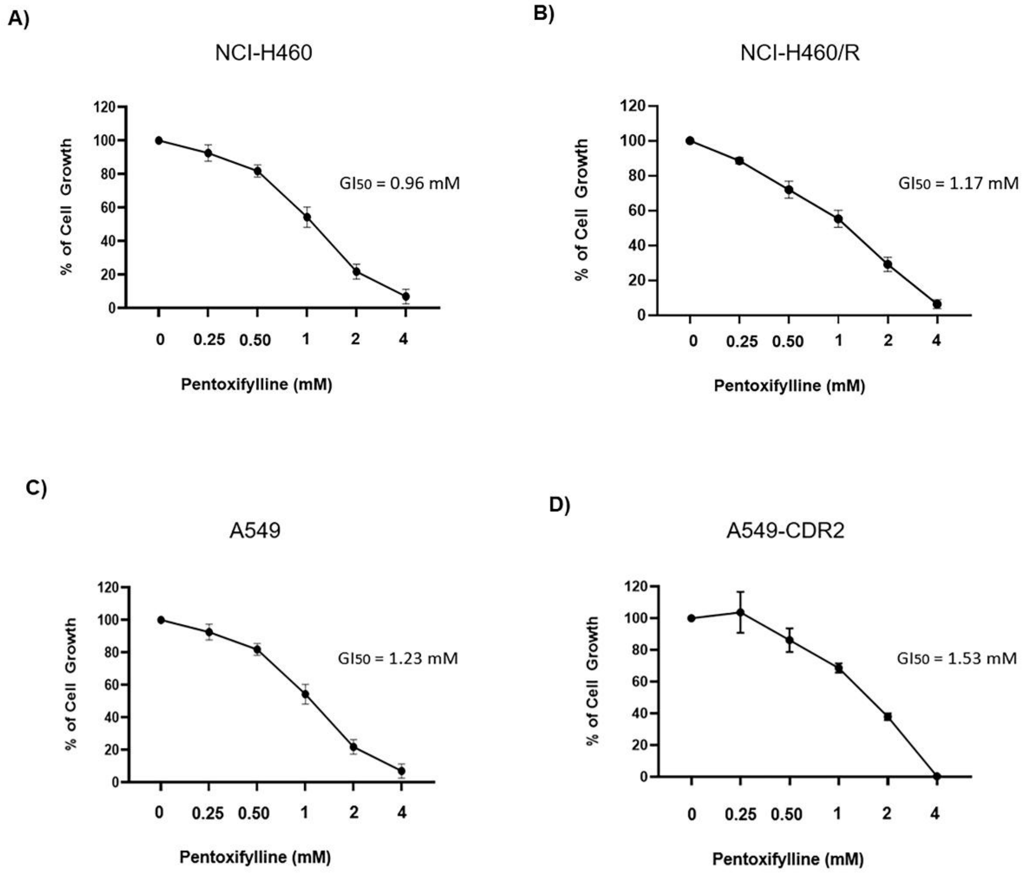

Supplementary Figure 2. Concentration-response curves of pentoxifylline on two NSCLC pairs of sensitive and MDR cell lines: (A) NCI-H460 *vs.* (B) NCI-H460/R; and (C) A549 *vs.* (D) A549-CDR2. Data is presented as percentage of cell growth and are the mean ± SEM of a minimum of three independent experiments.

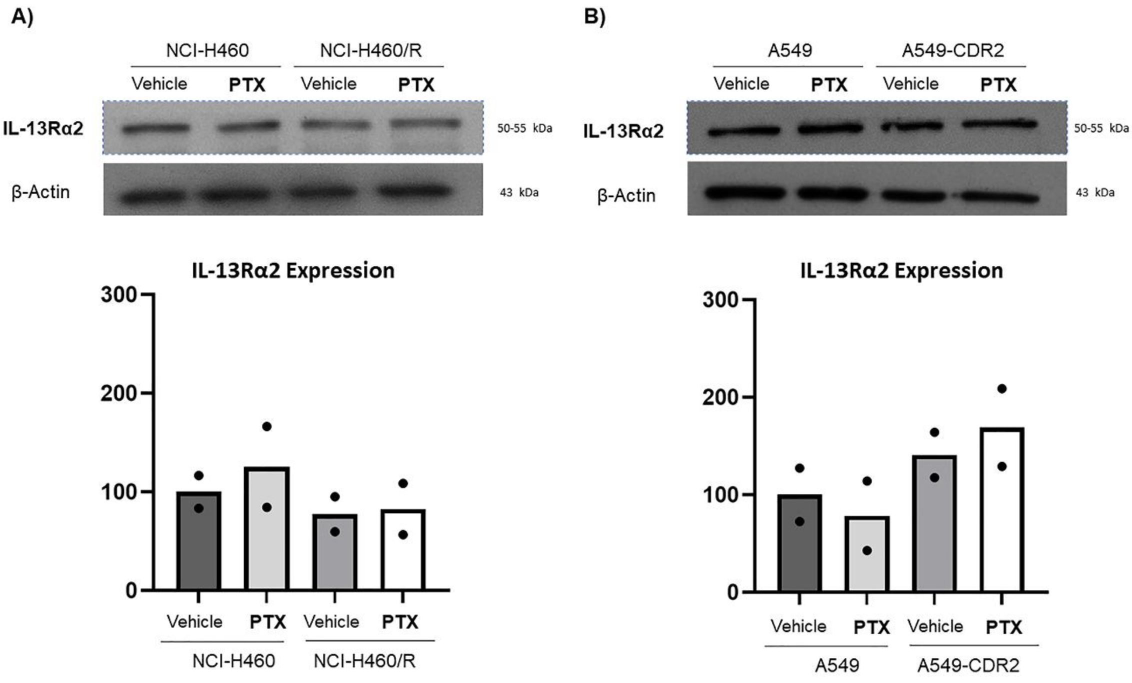

Supplementary Figure 3. Effect of pentoxifylline on the expression levels of IL-13Rα2, determined by Western Blot, on (A) NCI-H460 *vs.* NCI-H460/R and (B) A549 *vs.* A549-CDR2 cell lines, treated with vehicle (H_2O) or pentoxifylline at 1.0 mM after 48 h treatment. β-Actin was used as loading control. Data is from two independent experiments.

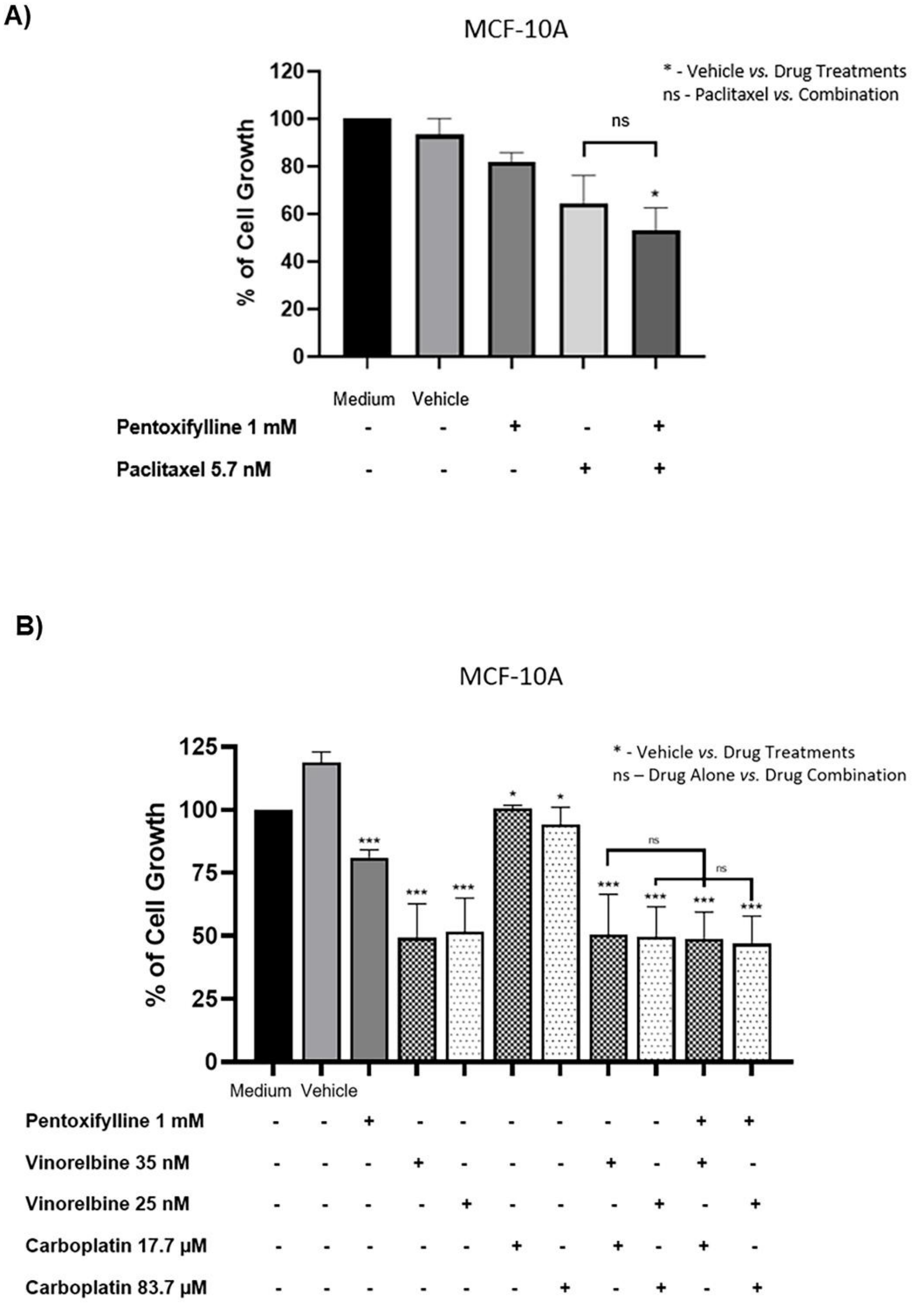

Supplementary Figure 4. Effect of the combined treatment consisting of pentoxifylline with (A) Paclitaxel and (B) Vinorelbine and/or Carboplatin, in the non-tumorigenic cell line MCF-10A, assessed by the SRB assay. Data is presented as a percentage of cell growth and are the mean ± SEM of a minimum of independent experiments. $^{*}P \leq 0.05$; $^{***}P < 0.001$; ns: $P > 0.05$.
